# Critical scale of propagation influences dynamics of waves in a model of excitable medium

**DOI:** 10.1186/1753-4631-3-4

**Published:** 2009-07-09

**Authors:** Joseph M Starobin, Christopher P Danford, Vivek Varadarajan, Andrei J Starobin, Vladimir N Polotski

**Affiliations:** 1 Department of Physics and Astronomy, University of North Carolina at Greensboro, Greensboro, NC, USA; 2 Mediwave Star Technology, Inc, Greensboro, NC, USA

## Abstract

**Background:**

Duration and speed of propagation of the pulse are essential factors for stability of excitation waves. We explore the propagation of excitation waves resulting from periodic stimulation of an excitable cable to determine the minimal stable pulse duration in a rate-dependent modification of a Chernyak-Starobin-Cohen reaction-diffusion model.

**Results:**

Various pacing rate dependent features of wave propagation were studied computationally and analytically. We demonstrated that the complexity of responses to stimulation and evolution of these responses from stable propagation to propagation block and alternans was determined by the proximity between the minimal level of the recovery variable and the critical excitation threshold for a stable solitary pulse.

**Conclusion:**

These results suggest that critical propagation of excitation waves determines conditions for transition to unstable rhythms in a way similar to unstable cardiac rhythms. Established conditions were suitably accurate regardless of rate dependent features and the magnitude of the slopes of restitution curves.

## Background

Studies of unstable waves in reaction-difusion systems are the subject of significant theoretical and practical importance, particularly with respect to the analysis of biological excitable media such as nerve and cardiac tissue [1,2]. Pioneering studies suggested that propagation of waves in these media may be governed by a few fundamental parameters which influence formation of the excitation wavefront [3,4]. One such parameter, ϵ = τ_*f*_/τ_*r *_≪ 1, reflects the two order-of-magnitude difference in time constants between the fast excitation, *τ*_*f*_, and slow recovery, *τ*_*r*_, processes, while another one, excitation threshold, *v*_*r*_, is linked to the critical level of excitation necessary to initiate the propagation of an excitation pulse through the medium.

Although examination of instabilities based on this approach would possess an obvious advantage of being theoretically analyzable, attention has primarily focused on experimentally motivated methods. It has been shown in tissue preparations that the duration of excitation, known as action potential duration, and the speeds of excitation wavefronts and wavebacks depend on previous stimulation periods and refractory (diastolic) intervals [5,6]. The analysis of such dependences, known as restitution curves, has been introduced as a method for evaluation of stability of excitation waves in biological excitable media [7-17].

Numerical simulations also confirmed these findings and demonstrated that *T*_*h *_and *T*_*f *_can be fitted to specific restitution curves (called a restitution portrait) using time dependent gate variables in various reaction (ionic) models [18-25]. Several recent studies implemented an approach based on a projection of different ionic models to discrete maps with sufficient rate-dependence (memory) and multi-component equivalent restitution portraits [20-27]. However, it was later confirmed that none of the stability criteria derived from these complex discrete maps predicted the onset of alternans in tissue studies [28]. Computational experiments described in [19] suggest that such deficiency is more likely to result from lack of analysis of propagation in the medium as a whole rather than from the lack of particular details of the ionic models of individual excitable cells.

To address this deficiency we introduce an alternative approach based on the analysis of a modified Chernyak-Starobin-Cohen (CSC) reaction-diffusion model which accounts for effects of wave propagation. In contrast to the complexity of the majority of existing reaction-diffusion models, the CSC model employs just a few fundamental parameters and is analytically solvable. It offers a rigorous criterion for determining the stability of excitation wave propagation in an excitable cable [29,30].

In this paper, we study the stability of excitation waves by examining their propagation in a one-dimensional CSC medium, and modify the model accordingly to incorporate the memory linked pacing rate driven adjustments of excitation threshold, *v*_*r*_. This modification was motivated by direct experimental measurements of *v*_*r *_that demonstrated that exponential-like evolution of excitation threshold takes place over the course of multiple pacing cycles following stepwise changes in pacing rate in guinea pig and human ventricular muscle [31-33]. Such an approach allows us to establish the condition for stability of propagation of excitation waves using analytical and numerical solutions of the reaction-diffusion model while incorporating into the model experimentally observed action potential restitution dynamics.

We demonstrate that in a wide range of excitation thresholds, *v*_*r*_, and pacing rates, *T*_*m*_, regardless of the particular values of slopes of restitution curves and rates of adaptation of *v*_*r *_(more or less memory), the loss of stability of waves in an excitable cable of finite length is determined by proximity of the minimal level of the recovery variable, *v*, at the foot of the action potential and the critical excitation threshold of the solitary pulse computed analytically in [29].

## Methods

Basic equations that describe a class of exactly solvable models for excitable media have been defined in [29,30]. Here we introduce a modification of this analytical model by adjusting the excitation threshold, *v*_*r*_, in response to changes in frequency of external pacing. We will consider the model in dimensionless form:

(1)

(2)

(3)

*u*(*x*, *t*) and *v*(*x*, *t*) are a membrane potential and slow recovery current, respectively. *λ*, ϵ, *ζ*, and *τ *are the model parameters, where *τ*^-1 ^< ϵ. The scaling of the system is described below.

The pacing function, *P *(*x, t*), is defined as a product of two functions, *X*(*x*) and *Y *(*t*). Each is composed of the Heaviside step function, Θ (*x*), as follows: *X*(*x*) = *A *[Θ(*x-δ*_1_) - Θ(*x-δ*_2_)] and *Y *(*t*) = Θ (*t*_*k*_) - Θ (*t*_*k *_+ *T*_*s*_), where *A *and *δ*_2 _- *δ*_1 _are the amplitude and width of the pulse, respectively, *T*_*s *_is the pulse duration, and *t*_*k *_= *t*_*N*(*m*-1) _+ [*k *- *N *(*m *- 1)]*T*_*m *_are the instants of time when stimuli are delivered. *T*_*m *_is the pacing period, *N *represents the number of stimuli at each pacing interval plateau, the index *m *denotes each pacing plateau, *m *= 1, ..., *M*, and *k *is an integer in the range *k *= 1, ..., *N M *. The number of stimuli *N *is the same for all plateaus. Overall during the course of the protocol, *T*_*m *_progressively decreases to the minimal value when stable propagation of the wave is still possible.

The right-hand term *B *in Eq. (3) responds to a stepwise evolution of pacing period, *T*_*m*_. Similar to the experimental findings described in [31,32], stepwise changes in *B *result in smooth exponential transition of the excitation threshold from one steady-state plateau to another. For the sake of simplicity, a steady-state value of excitation threshold at each *m*^th ^plateau, , was chosen to be linearly dependent on the corresponding pacing interval, *T*_*m*_:

(4)

Here *α *and *β *are positive parameters that determine the amplitude of change of *B*_*m *_between two consecutive pacing plateaus.

The scale of *u *is the maximum steady-state action potential amplitude *U*_0_, the scale of *v *is given by *σ*_*NA*_*U*_0_, and the time scale is *C*_*m*_/*σ*_*NA*_, where *σ*_*NA *_corresponds to the maximum sodium conductance and *C*_*m *_is the membrane capacitance. The characteristic length scale is given by , where *D *is the diffusion coefficient. The small parameter ϵ ≪ 1, is equal to *C*_*m*_/(*t*_*K*_*σ*_*NA*_) and *ζ *= *σ*_*K*_/*σ*_*NA *_where *σ*_*K *_and *τ*_*K *_correspond to the maximum potassium conductance and potassium current time constant, respectively.

## Results and discussion

**Figure 1:**
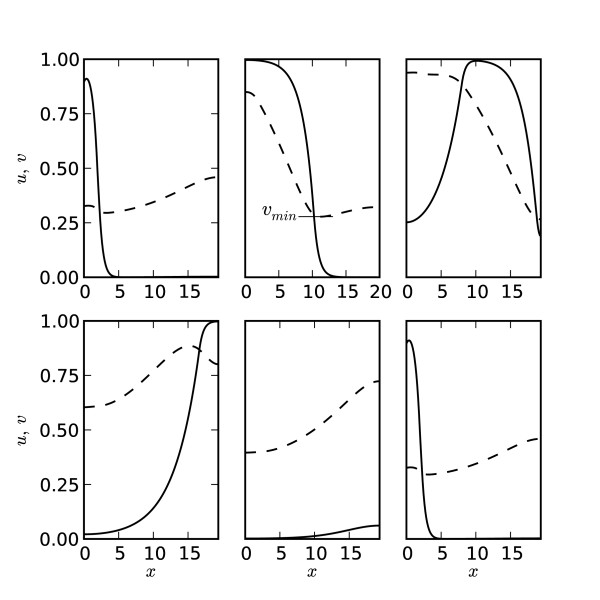
**Formation and propagation of a pulse**. Formation and propagation of a pulse between successive pacing stimuli applied at the left end of the cable (v_*r *_= 0.19, *v*_*min *_= 0.29, and *T*_*h *_= 9.0 measured at *x*_0 _= 10). Solid and dashed lines show spatio-temporal dynamics of *u *and *v*, respectively. The first and last snapshots correspond to the moments when two successive pacing stimuli are applied. Time intervals between the snapshots are equal to 7.2 (20% of the pacing period).

The system of Eqs. (1)–(3) was solved numerically on a short cable of 150 grid points with spatial and temporal grid intervals of Δ*x *= 0.13 and Δ*t *= 7.2 × 10^-4^, respectively. Periodic wavetrains were produced by stimulating the cable with a square wave at the left end using the function *P *(*x, t*) = *X*(*x*)*Y *(*t*), defined above, where *A *= 1.2, *δ*_1 _= 2Δ*x*, *δ*_2 _= 15Δ*x*, and *T*_*s *_= 10^3^Δ*t*. The model parameters *λ*, ϵ, and *ζ *were equal to 0.4, 0.1, and 1.2, respectively, for all simulations. Numerical solutions were computed using a second-order explicit-difference scheme with no-flux boundary conditions [13]. A typical solution as shown in Figure 1 depicts the propagation of a single pulse between two successive pacing stimuli. The length of the cable is approximately equal to the width of the fully developed pulse to reflect the physiologically relevant relative dimensions of the heart and a propagating excitation wave in a wide range of heart rates and excitation thresholds.

In order to quantify the dynamics of the system in response to perturbations in *T*_*m *_(Eqs. (1)–(3)), we computed the action potential duration (*T*_*h*_), the diastolic interval (*T*_*f*_) at each pacing period, and the minimal value of the recovery variable at the foot of the propagating pulse, *v*_*min *_(Figure 1). The values of *v*_*min *_are always greater than *v*_*r *_due to incomplete medium recovery from repeated pacing, and *v*_*min *_increases monotonically with decreasing *T*_*m*_. The action potential duration was defined as the interval of time when *u *> *v *at a specified node, *x*_0_. Accordingly, the refractory period, *T*_*f*_, was defined as the interval of time when *u *<*v*. In order to analyze steady-state duration of the developed pulse, we measured *T*_*h *_at the center of the cable *x*_0 _= 10.

### Stability of propagation for constant excitation threshold

**Figure 2:**
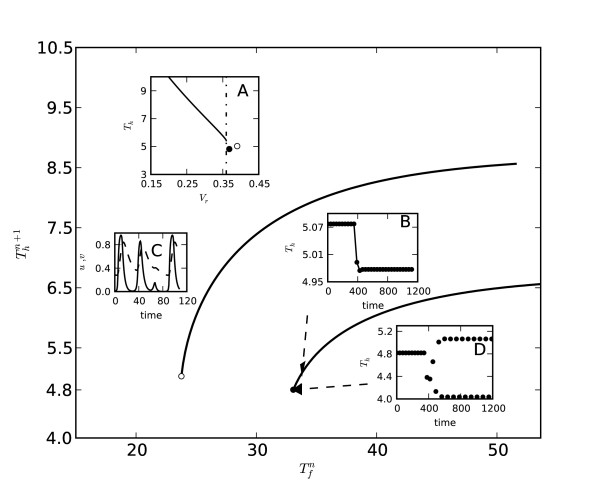
**Steady state restitution curves with constant *v*_*r*_**. Steady state restitution curves for two values of excitation threshold v_*r *_= 0.25 (upper curve) and v_*r *_= 0.31 (lower curve). Insert A shows the analytically determined dependence of *T*_*h *_on excitation threshold v_*r *_for stable solitary pulse [29]. Critical level of  = 0.359 beyond which no stable solitary pulse exists is indicated by the dashed line. Open and filled circles located near the intersection above and below horizontal dashed line correspond to the ends of restitution curves shown as solid lines in the main domain. Inserts B and D illustrate the transition between stable action potential adaptation and alternans at the end of the curve with higher value v_*r *_= 0.31. Insert C shows the evolution of *u *and *v *during a 3:2 propagation block at the end of the curve with lower value v_*r *_= 0.25.

The system of equations was initially studied with Eq. (3) replaced by its asymptotic form, which is equivalent to *τ *= ∞. The cable was stimulated periodically in a stepwise manner with one percent decrements between each pacing plateau (40 consecutive pacing periods) over the range *T*_*m *_= 61 to 28. At the end of each plateau, *T*_*h*_, *T*_*f*_, and *v*_*min *_had all reached steady state values that were used to compose the steady state restitution curve and determine the minimal stable action potential duration, , for a given excitation threshold. Steady state restitution curves computed for two different excitation thresholds are shown in Figure 2.

Insert A portrays the analytical dependence of *T*_*h *_on excitation threshold *v*_*r *_for a stable solitary pulse [29]. It also shows the critical level of excitation threshold ( = 0.359, vertical dashed line) beyond which no stable solitary pulse exists. For the repeatedly paced cable, the numerically determined minimal steady state values, , at the ends of the two steady-state restitution curves are depicted by the two circles (open and filled) in the lower right corner of insert A, which are to the right of the vertical dashed line *v *=  (Figure 2). The location of both circles demonstrates that the loss of stability for the sequence of pulses occurs when *v*_*min *_is close to the critical excitation threshold for the stable solitary pulse . The character of the loss of stability at the ends of restitution curves depends on the margin between *v*_*min *_and  which decreases for greater values of excitation threshold *v*_*r *_(Figure 2, Insert A, filled circle).

It was observed that for = 0.1 complexity of block type behavior for higher values of *v*_*r *_evolved from 2:1 to 3:2 responses, followed by *T*_*h *_alternans when the difference between *v*_*min *_and  was less than 3%. The evolution from stable propagation to propagation block and *T*_*h *_alternans is illustrated by inserts B, C, and D, respectively. Insert B characterizes stable adaptation of *T*_*h *_from one steady-state to another. The adaptation is rapid, and as a result the steady-state and S1–S2 curves nearly coincide. At the end of the restitution curve with lower values of *v*_*r *_stable action potential propagation transforms into a block type N:M (N>M) response shown in insert C (upper curve, *v*_*r *_= 0.25). For higher values *v*_*r *_when *v*_*min *_is closer to , stable propagation transforms to *T*_*h *_alternans shown in insert D (lower curve, *v*_*r *_= 0.31).

Similar trends were observed for higher and lower values of ϵ. For ϵ = 0.06 2:1 conduction blocks transformed directly to alternans regardless of the difference between  and *v*_*min*_. For ϵ = 0.14 however, there was a progression from 2:1 responses to more complex 3:2 patterns which culminated in alternans as the difference between  and *v*_*min *_decreased from approximately sixteen to one percent.

Obtained results confirm that irrespective of the restitution slopes, alternans do not appear until, at the end of the restitution curve, the value of *v*_*min *_exceeds  by some small margin (Figure 2). Under these conditions alternans appear for slopes smaller than one (*S *= 0.44, Figure 2 lower curve), and conversely, stable propagation occurs for slopes greater than one (*S *= 1.94, Figure 2 upper curve).

Finally, although very useful for stability analysis, the original CSC model with constant excitation threshold does not allow reconstruction of transitional restitution behavior near a change in pacing rate. Appropriate model enhancements are described in the next section.

### Stability of propagation with rate-dependent *v*_*r*_

In order to reproduce experimentally observed restitution curves, the system of equations (1)–(2) was upgraded with Eq. (3), which allowed us to incorporate effects of memory [28]. We will show that in this system the criterion for the appearance of action potential duration alternans will be the same as in the memory-less case detailed in the previous section describing the cable with constant excitation threshold.

The pacing protocol described previously was used again to construct the steady-state restitution curve and determine the minimal stable action potential durations. In addition, a similar stepwise protocol with larger 5% decrements in *T*_*m *_was used. A conventional S1–S2 restitution protocol, in which the cable was stimulated for forty consecutive periods with a given *T*_*m *_(S1) followed by delivery of a premature S2 stimulus, was used to compute S1–S2 curves around given steady-state values of *T*_*h*_.

A gradual phase of constant BCL adaptation followed the immediate S1–S2 response to an abrupt change in pacing rate, and the *T*_*h *_transients had a duration of 5–50 stimulation periods depending on the adaptation constant, *τ *. Pictured in the restitution domain, this combination of S1–S2 and constant BCL responses formed the triangles typical of experimental and computational studies of ventricular action potential [21,22,24].

**Figure 3:**
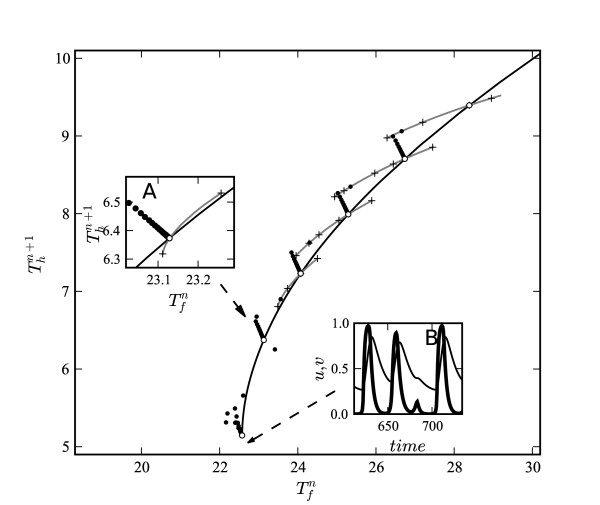
**Restitution curves**. A series of S1–S2 and constant BCL restitution curves superimposed on the dynamic restitution curve for *β *= 5 × 10^-3 ^and *α *= 0.375. The range of excitation thresholds depicted, therefore, is from *v*_*r *_= 0.18 at *T*_*m *_= 37.7 to v_*r *_= 0.23 at *T*_*m *_= 27.7. The insert shows a close-up view of the S1–S2 restitution curve around the point *T*_*m *_= 29.4 where the S1–S2 curve is steeper than the dynamic restitution curve.

Figure 3 shows a superposition of the steady state restitution curve with five transitions between different steady-state values of *T*_*h *_computed with 5% changes in *T*_*m *_starting with *T*_*m *_= 37.7 (*β *= 5 × 10^-3^, *α *= 0.375). At longer BCLs, the S1–S2 curves are shallower than the steady-state restitution curves, and all of the transient constant BCL responses after the first response to the change in stimulation rate lie on a straight line with negative slope, which is referred to as the "BCL-line" [21,22,28]. At shorter BCLs, the slope of the S1–S2 curve increases. When the slope of the S1–S2 curve (insert A) is greater than the slope of the steady-state curve, the response (dots near the end of steady-state curve) to a decrease in stimulation interval evolves as a series of oscillations that damp to a new steady-state value.

**Figure 4:**
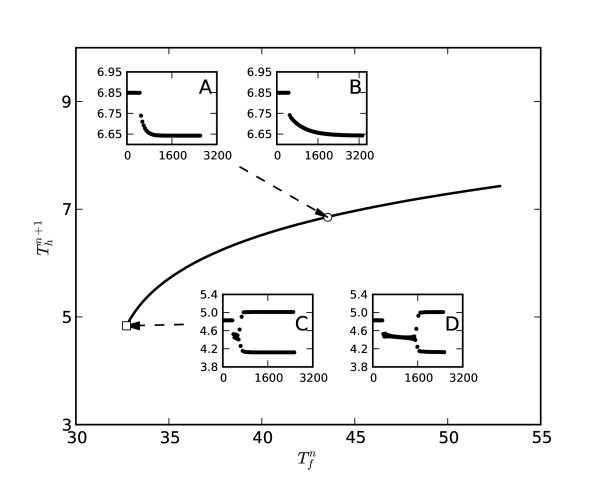
**Development of alternans**. Steady-state restitution curve for rate-dependent excitation threshold (*β *= 1 × 10^-3 ^and *α *= 0.346). Inserts A and B show *T*_*h *_response to a 5% perturbation in stimulation rate, for initial *T*_*m *_= 50.40 (v_*r *_= 0.30). The adaptation constant for insert A is *τ *= 144 and for insert B is *τ *= 576. Inserts C and D show *T*_*h *_alternans for the same two adaptation constants (*τ *= 144 for C and *τ *= 576 for D) with initial *T*_*m *_= 37.94 (v_*r *_= 0.31). The horizontal dashed line near the end of the restitution curve marks the critical duration of a solitary pulse, .

Figure 4 summarizes some typical *T*_*h *_responses to an abrupt change in pacing rate. In inserts A and B, the response is a gradual adaptation to a subsequent steady-state during which time the BCL is constant. The duration of this adaptation is directly controlled by the constant, *τ*. When *τ *is small, the bifurcation occurs almost immediately after five stimulation periods (Figure 4, insert C). When *τ *is large, the bifurcation is correspondingly delayed as shown in the insert D. This might be understood as "sliding" into the unstable area after an abrupt change in pacing rate due to the gradual increase of *v*_*r*_, rather than simply jumping into it as for the cable with constant excitation threshold.

Our simulations showed that complexity of wave dynamics for the system (1)–(3) was also determined by the difference between the values of *v*_*min *_and critical excitation threshold for a stable solitary pulse . For ϵ = 0.1 at the end of the restitution curve for the case shown in Figure 3 the value of *v*_*min *_was 10% greater than the value of . Under these circumstances, we found that damping oscillations preceded conduction block type 3:2 responses (Figure 3, insert B). When the difference between *v*_*min *_and  decreased to 3% of , perturbations of *T*_*m *_transformed into persistent *T*_*h *_alternans (Table 1).

**Table 1:** Evolution of Wave Dynamics.

ϵ = 0.06, = 0.40			ϵ = 0.10, = 0.36			ϵ = 0.14, = 0.33		
*v*_*r*_	Type	%*	*v*_*r*_	Type	%*	*v*_*r*_	Type	%*

0.12	2:1	10.75	0.19	2:1	9.47	0.15	2:1	16.36

0.16	2:1	7.50	0.21	2:1	10.30	0.19	3:2	14.24

0.20	2:1	3.25	0.25	3:2	8.63	0.23	3:2	10.60

0.27	2:1	2.00	0.27	4:3	7.77	0.27	2:2	6.67

0.33	2:2	1.25	0.31	2:2	2.78	0.29	2:2	1.81

The types of wave dynamics near the ends of steady-state restitution curves for different values of ϵ and rate dependent excitation threshold *τ *= 172, and

Similar to simulations with constant *v*_*r*_, for rate dependent excitation threshold the complexity of transition from blocks to alternans also increased for higher values of parameter ϵ. For ϵ = 0.06 transformation from 2:1 blocks to alternans was almost abrupt. However, for higher values of ϵ as the difference between *v*_*min *_and  further decreased, the transition to alternans was more complex and evolved through stages with higher 3:2 and 4:3 complexity of propagation blocks (Table 1).

## Conclusion

Previous attempts to determine conditions for action potential duration alternans have focused on the analysis of the slopes of restitution curves in various theoretical and experimental models. However, these efforts did not result in a consistent theoretical criterion for prediction of action potential duration instabilities [22,24,27,28]. In part this happened because stability was examined solely in regard to the magnitudes of particular restitution slopes rather than with the analysis of the conditions for a critically propagating pulse [13,34,35].

In this paper, we have demonstrated that stable propagation of excitation waves in a paced cable occurs until the minimal level of the recovery variable in front of the rising action potential wave reaches a value that is greater than the critical excitation threshold for the stable solitary pulse by some small margin. This margin decreases for higher *v*_*r*_, which agrees with previous analytical findings for the CSC model approximating the critical speeds of an infinite wavetrain [30]. At the limit of the critically stable pulses, our numerical system revealed two types of unstable behavior. When the minimal value of recovery variable at the foot of the propagating wave approached the critical level of excitation threshold beyond which no stable solitary pulse was able to propagate, we observed conduction blocks of increasing complexity followed by alternans. Alternans developed slower for larger values of the adaptation constant, *τ*.

It was demonstrated that these conditions were valid with or without rate-dependent features and regardless of the magnitude of the slopes of restitution curves.

## Competing interests

JS is Chief Science Officer of Mediwave Star Technology, Inc.

## Authors' contributions

JS, CD, VV, AS, and VP conceived and designed the theory and numerical studies. CD, VV, and AS performed the numerical simulations. JS, CD, VV, AS, and VP analyzed the results. JS, CD, VV, AS, and VP compiled and the manuscript.
